# Protein Sources Alternative to Meat: State of the Art and Involvement of Fermentation

**DOI:** 10.3390/foods11142065

**Published:** 2022-07-12

**Authors:** Mariagrazia Molfetta, Etiele G. Morais, Luisa Barreira, Giovanni Luigi Bruno, Francesco Porcelli, Eric Dugat-Bony, Pascal Bonnarme, Fabio Minervini

**Affiliations:** 1 Dipartimento di Scienze del Suolo, della Pianta e degli Alimenti, Università degli Studi di Bari Aldo Moro, Via Amendola 165/a, 70126 Bari, Italy; mariagrazia.molfetta@gmail.com (M.M.); giovanniluigi.bruno@uniba.it (G.L.B.); francesco.porcelli@uniba.it (F.P.); 2 Centro de Ciências do Mar (CCMAR), Universidade do Algarve, 8005-139 Faro, Portugal; etiele@gmail.com (E.G.M.); lbarreir@ualg.pt (L.B.); 3 UMR SayFood, INRAE, AgroParisTech, Université Paris-Saclay, Avenue Lucien Brétignières, 78850 Thiverval-Grignon, France; eric.dugat-bony@inrae.fr (E.D.-B.); pascal.bonnarme@inrae.fr (P.B.)

**Keywords:** meat-alternative proteins, fermentation, amino acid profile, single-cell proteins, edible mushrooms, microalgae, cereals, pseudocereals, legumes, insects

## Abstract

Meat represents an important protein source, even in developing countries, but its production is scarcely sustainable, and its excessive consumption poses health issues. An increasing number of Western consumers would replace, at least partially, meat with alternative protein sources. This review aims at: (i) depicting nutritional, functional, sensory traits, and critical issues of single-cell proteins (SCP), filamentous fungi, microalgae, vegetables (alone or mixed with milk), and insects and (ii) displaying how fermentation could improve their quality, to facilitate their use as food items/ingredients/supplements. Production of SCP (yeasts, filamentous fungi, microalgae) does not need arable land and potable water and can run continuously, also using wastes and byproducts. Some filamentous fungi are also consumed as edible mushrooms, and others are involved in the fermentation of traditional vegetable-based foods. Cereals, pseudocereals, and legumes may be combined to offer an almost complete amino acid profile. Fermentation of such vegetables, even in combination with milk-based products (e.g., tarhana), could increase nutrient concentrations, including essential amino acids, and improve sensory traits. Different insects could be used, as such or, to increase their acceptability, as ingredient of foods (e.g., pasta). However, insects as a protein source face with safety concerns, cultural constraints, and a lack of international regulatory framework.

## 1. Introduction

The world population is estimated to reach ca. 10 billion people by 2050 [1] implying the need to produce a higher amount of food than today [2]. Among different food items, some products of animal origin, such as meat and dairy products, deplete natural resources at a higher degree than food of vegetable origin (1 kg of meat or milk requires the use of 7 kg of vegetables) [3]. Yet, such food products represent important sources of dietary proteins and other micronutrients. In Europe (2019), the consumption of food of animal origin provided ca. 55% of total protein daily intake [4]. Cereals are the main source of protein intake (ca. 32%), followed by meat (ca. 26%), dairy products (ca. 20%), fish and seafood (ca. 6%), fruits and vegetables (ca. 5%), eggs (ca. 4%), and pulses (ca. 2%). Other food items, such as tree nuts, sum up to 100% [5]. Moreover, an increasing number of people living in developing countries are gaining access to meat and dairy products. Estimates dating back to 2010 envisage that dramatic increases in the demand for meat (+173%) and dairy products (+158%) will occur by 2050 [6].

Meat production and consumption, besides having a huge ecological footprint, pose other serious issues, especially related to health. For instance, associations were reported between consumption of red and processed meats and increased risk of developing colon, breast, pancreas, and prostate cancers [7,8,9,10,11,12,13] and high prevalence of chronic diseases [8,11,14]. Although the exact mechanisms underlying the association between meat consumption and risk of cancer development are still to be elucidated, some studies suggest that red or processed meat increases the number of preneoplastic lesions [9]. Some genotoxic compounds (e.g., nitroso-compounds, heterocyclic aromatic amines) in meat lead to mutations of the adenomatous polyposis coli gene. Other meat compounds (e.g., heme) in meat increase oxidative stress [9]. In addition, consumption of meat carries an inherent risk of contracting zoonosis and/or being exposed to antibiotics and hormones used in animal breeding [8,15]. Therefore, based on concerns about sustainability of meat production, animal welfare, and health issues associated with excessive meat consumption, an increasing number of consumers in the Western world (USA and other developed countries) would replace, at least partially, meat with other dietary proteins sources [16]. In this perspective, in 2019, a consortium of researchers from 42 universities or research centers located in 8 European countries launched the SYSTEMIC project (an integrated approach to the challenge of sustainable food systems: adaptive and mitigatory strategies to address climate change and malnutrition). The project aims to develop pathways for a food system transformation, which is climate-resilient and able to cope with societal challenges. It is providing information on proven options that provide sustainable and nutritious food, such as alternative protein-rich foods (e.g., vegetables, filamentous fungi, algae, microalgae and other microorganisms, insects, and “cultured” meat) that can possibly replace meat [17].

Since 2019, some interesting reviews have focused on different features of dietary protein sources that represent alternatives to meat [15,16,18,19,20,21]. However, to our knowledge, no article has considered the potential of combining alternative protein sources and fermentation. Thus, this review aims at: (i) depicting nutritional, functional, sensory traits, and critical issues of single-cell proteins, filamentous fungi, microalgae, vegetables (alone or mixed with milk), and insects and (ii) displaying how fermentation could contribute to improve the quality aspects of these alternative protein sources, to facilitate their use as food items/ingredients/supplements providing consumers with dietary proteins alternative to meat. The food industry could benefit from understanding the advantages of applying fermentation to alternative protein sources.

## 2. Single-Cell Protein as a Source of Dietary Proteins

Single-cell protein (SCP) often refers to edible microbial biomass produced from single-cell microorganisms such as bacteria, archaea, fungi (including yeasts), and some algae [22]. The use of SCPs for human consumption has many advantages compared to animal or plant proteins. First, SCP production is not dependent on agriculture or climate. Hence, it is not subjected to seasonal variations, and does not need arable land and potable water. Furthermore, it can be virtually installed everywhere and can potentially run without interruption [23,24]. Second, microorganisms can grow on a wide array of substrates including inexpensive wastes (e.g., food wastes or food processing byproducts, wastewaters, agricultural and forestry wastes), CO_2_, or methane [25,26,27]. SCPs production is therefore considered as an environment-friendly process and microbial biomass represents a credible alternative solution to ensure future food security while minimizing the impact on the global sustainability [26,28].

### 2.1. General Characteristics of SCP

Although highly concentrated in proteins—up to 80% on a dry matter basis [25]—SCPs also usually contain lipids, carbohydrates, and several vitamins and minerals [23,29]. SCPs are considered to have a high nutrititional value compared to animal or plant proteins [22,29,30,31]. Until now, only a limited number of microorganisms have been used to produce SCPs for human consumption (listed in [22,25]). These microorganisms mostly comprised algae, e.g., *Arthrospira platensis* and *Chorella vulgaris* (more details available in Section 4 of this review), and fungi such as *Saccharomyces cerevisiae*, *Cyberlindnera jadinii* (formerly *Pichia jadinii* or *Candida utilis*) and *Fusarium venenatum*. Bacterial SCPs are also commercially available, mostly for feed applications until now but certainly for food in the future [25]. In particular, autotrophic bacteria, which can assimilate CO_2_ directly as a carbon source, are currently gaining much attention [27,32,33,34].

The protein content of SCP largely depends on the considered microbial species [26]. Growth conditions, including the substrate used for microbial growth, can also strongly affect the microbial metabolism and, consequently, the characteristics of SCP (e.g., protein content, amino acid profile) [35]. However, as a general rule, one can expect to produce SCP with 50–80% proteins from bacteria and 20–60% from yeasts [25,32,33].

As for protein content, the amino acids composition of SCP depends on the considered microorganism. However, it can be easily modulated by supplementing the culture media with organic or inorganic nitrogen sources. For instance, substantial variations of the amino acids composition of SCP produced from *Cyberlindnera jadini* FMJ12 using mango waste as the principal substrate were observed after supplementation of the growth media with yeast extract, peptone, ammonium sulphate, and ammonium nitrate [35]. Indeed, it is well established in the literature that nitrogen sources affect the metabolism of yeasts, including the synthesis of amino acids [36].

In 2000, Anupama and Ravindra attempted to rank SCP products from algae, fungi, and bacteria based on their average nutrititional values and production constraints [22]. They classified by order of preference, algae > fungi > bacteria, but highlighted the fact that the yield of many important components varies depending on the type of substrate used, the specific organism used, and the culture conditions.

One of the main defects of bacterial and yeast SCPs is their high nucleic acids (NA) content, which is 6 to 12% dry weight [32]. Ingestion of high amounts of NA by humans can lead to uric acid precipitation and, consequently, be responsible for health issues such as gout symptoms or kidney stones. Consequently, the production of SCPs from these microorganisms for food applications often requires an additional treatment (e.g., thermal, chemical, or enzymatic) for reducing the NA content below 2% dry weight [34]. This can be achieved by heat treatment at 60–70 °C [37,38], chemical treatment by using either NaCl, HCl, or NaOH [37,38], or by enzymatic treatment using ribonuclease and endonuclease [39].

### 2.2. Use of SCP from Yeasts and Bacteria in Food Production

Several products based on microbial biomass are available on the market of food and feed. In 2016, Ritala et al. [25] listed more than 40 companies involved in SCPs production. Regarding human consumption, SCPs are used directly as food products or as food ingredients, flavor ingredients, or nutraceuticals.

Few examples of the functional characterization of SCPs are available in the literature. SCP from *Saccharomyces cerevisiae* exhibits interesting functional properties, such as low bulk density, high water and oil absorption capacity, excellent foaming, and emulsifying properties. This makes *S. cerevisiae* SCP suitable for use as an ingredient for food such as baked goods, desserts, and sauces [40]. Rheological analyses showed that gels made with SCPs from kefir microorganisms (yeasts and lactic acid bacteria) grown on whey exhibit a harder texture profile than gels made with soy flour, probably due to their higher protein concentration [41]. Solein^®^, a bacterial SCP commercialized by Solar Foods company, showed comparable properties to pea protein isolate in terms of water and oil binding properties, foaming, and emulsifying capacity [23] and could thus, theoretically, serve as a basic ingredient for various food products such as baked goods, pasta, yogurt, or even microbial-based meat products. Sensory evaluation of SCPs is still lacking but it should be performed before application as food or food ingredient to evaluate consumer acceptability.

SCP could be exploited as cell factories for animal and vegetable proteins, through “precision fermentation”. This fine approach diverts resources so that microbial cell factories produce the desired protein, such as casein. A metabolically engineered yeast (*Komagataella pastoris*, homotypic synonym: *Pichia pastoris*) produces soy leg-hemoglobin, which imparts the color and flavor of meat to vegetable-based burgers [42]. Apart from that, fermentation could be effective for improving some characteristics of the final product such as taste, aspect, or digestibility. For instance, the use of microorganisms or the design of microbial consortia with desired bioconverting properties (e.g., proteolysis, aromatization through amino acids catabolism), could be a very efficient way to render SCP more acceptable. Such an approach is currently being developed on pea protein-based fermented products to reduce the “green note/beany”, and/or to develop new tastes (e.g., fruity notes, dairy-like notes) more acceptable to consumers [43,44]. The amino acid content of SCP [22], which is rich in methionine, could also be beneficially used to generate volatile sulfur compounds (VSCs) to produce cheese-like aromatized fermented products [45]) which could give familiar sensorial attributes to well-appreciated fermented dairy products.

## 3. Filamentous Fungi Are Invaluable Sources of Dietary Proteins

Filamentous fungi are used to produce a wide array of food ingredients (e.g., citric, gluconic, fumaric, kojic, itaconic acids, enzymes), nutraceuticals, terpenes, alkaloids, sesquiterpenoids, antibiotics (including penicillin, streptomycin, cephalosporin, griseofulvin, neomycin, tetracycline, vancomycin, gentamicin, rifamycin), pharmaceuticals (immunosuppressants, statins, contraceptives, ergot alkaloids, griseofulvin, lovastatin, taxol, zeranol), as well as animal feed and compounds used in agriculture, such as mycoherbicides and bio-fungicides [46,47,48,49]. They provide dietary proteins essentially in the form of edible mushrooms, mycoproteins, and traditional food obtained using fungi as microbial starters. The ascoma produced by truffles and the well-known basidiomata with a stalk and a cap (e.g., champignon, *Pleurotus* spp., shitake) are well-known examples of edible mushrooms [50,51,52,53]. Compared with plant- and animal-derived proteins, fungal-derived products show different nutritional profiles (Table 1), lower production costs, and higher environmental benefits. Indeed, filamentous fungi can be cultured on cheap substrates, usually consisting of byproducts and wastes from food processing (e.g., sugarcane bagasse for culturing *Penicillium janthinellum*), as well as from forestry and agricultural activities [54,55,56]. In addition, fermentation using edible filamentous fungi is a strategy to improve the nutrititional values of food. Mycoproteins production, as an alternative to plant- or animal-based food production, has important environmental advantages associated with (i) low environmental degradation, (ii) reduction/re-utilizing agri-food wastes, (iii) decentralized manufacturing, and (iv) reduction of greenhouse gas (GHG) emissions [56,57].

The protein content of filamentous fungi ranges from 10 to 63% and depends on the species and substrate of cultivation (Table 2). Filamentous fungi may grow as either mycelial when cultured on the surface of solid media, or diffuse mycelia or as dense pellets when grown in submerged cultures.

Edible mushrooms can be ingested as part of a well-balanced diet. They are especially rich in water and provide proteins, dietary fiber (insoluble “fibrous β-glucans matrix”), chitin (poly-N-acetyl glucosamine), vitamins (folate and B12), and some minerals (e.g., enough calcium, phosphorous, magnesium, zinc, selenium, but a low quantity of sodium). In contrast, they are poor in fat and have a limited caloric value (Supplementary Table S1). Edible mushrooms have a low degree of allergenicity [58,59,60,61,62,63,64].

Mycoprotein indicates a food product consisting of fungal biomass produced from fermentation [57]. The first mycoprotein was obtained using a selected strain of *Fusarium venenatum* cultured on a glucose-rich substrate [54]. Mycoproteins include 50–55% of proteins, which are rich in lysine, but poor in cysteine and methionine [56]. Glutathione, thiamine, folic acid, and riboflavin are other nutritionally interesting molecules contained in mycoproteins [26,57]. Mycoproteins offer a favorable fatty acid profile and high fiber content. Fat in mycoproteins is represented by polyunsaturated fatty acids (e.g., linoleic and linolenic acids), while being cholesterol-free, because fungal sterols differ from those of animals [63]. However, compared to animal-sourced proteins, mycoproteins contain lower levels of vitamin B12 and iron [55,56]. Toxicology studies showed that long- or short-term consumption of mycoproteins exerts no adverse effects on the normal growth of humans and animals. Furthermore, the risk of sensitization or intolerance to mycoproteins is exceptionally lower than for many other common foods (e.g., milk, soy, legumes, crustacean shellfish, and eggs) [25,56,58,63]. Food-grade mycoprotein derived from non-crop feedstocks (e.g., lignocellulosic agricultural residues such as rice straw) offers potential sustainable solutions and remains an open area for investigation [57].

During 1950–1970, mycelium of filamentous fungi was largely used to produce SCP for human and livestock consumption [22,25,65]. Fungal SCP with a complete amino acids profile [66,67] can be produced starting from starch or byproducts, such as thin stillage, starch plant wastewater, spent liquors from pulping processes, and vinasses [67,68]. Technically, fungal SCP is obtained by culturing filamentous fungi in submerged or solid-state fermentation process. After fermentation, biomass is harvested and may be subjected to washing, cell disruption, protein extraction, and purification. Baker’s yeast wastewater, pea-, and starch-processing byproducts are feedstock materials suitable to produce SPCs of *Aspergillus oryzae*, *Monascus purpureus*, *F. venenatum*, *Neurospora intermedia*, *Rhizopus arrhizus* (syn: *Rhizopus oryzae*), *Mucor indicus*, *Mucor hiemalis*, or *Rhizopus microsporus* (syn: *Rhizopus oligosporus*), with mycoproteins in the range 35–55% [25,57].

### 3.1. Fermented Foods Obtained Using Filamentous Fungi

Fermented foods such as mold-ripened meat and cheese are widespread in Europe and North America. For instance, blue-veined cheeses are made by using *Penicillium roqueforti* [54,57]. However, most fermented foods obtained using filamentous fungi as microbial starters have a millennia-long tradition in the east and southeast Asia.

Koji and the liquid condiment soy sauce are Japanese, Korean, and Chinese fermented foods produced by inoculating koji starter (consisting of koji fungi conidia) onto a steamed cereal grain (rice, barley, soybean, wheat, and other cereals) under opportune conditions of temperature and humidity. The koji fungi used as a starter are *A. oryzae*, *Aspergillus sojae* and several species included in the Black Aspergilli group, namely *Aspergillus luchuensis* (syn. *Aspergillus luchuensis* var. *awamori*), and its albino mutant *Aspergillus luchuensis* mut. *kawachii* (*Aspergillus kawachii*) [55,57,68,69,70]. *A. oryzae*, also referred to as koji mold, is also used to produce alcoholic beverages such as sake or shocho and condiments such as mirin [57,70,71]. Sake is a traditional Japanese fermented alcoholic drink that is brewed using *A. oryzae* to degrade the starch from rice into sugar, followed by yeast fermentation to produce ethanol [71]. *A. oryzae* is also used to ferment oilcake, a byproduct of pressing oil from soybeans, sunflower seeds, rapeseed, and peanut seeds, leading to increased protein content and decreased (73%) concentration of lipids [71]. Indeed, filamentous fungi improve nutritional values of the cultivation substrates, thanks to their proteolytic enzymes releasing amino acids that are partly incorporated in the fungal biomass. In addition, they synthesize enzymes that alter the distribution pattern of fatty acids and degrade antinutritional compounds [70].

Miso is a traditional Japanese seasoning of semisolid soybean and/or grain-based paste produced upon fermentation with *A. oryzae*. Four types of miso are produced depending on the ingredients used. Rice miso combines rice fermented with koji mold, soybeans, and salt. Barley miso mixes barley or naked barley fermented with koji mold, soybeans, and salt. Soybean miso blends soybeans, koji, and salt. Mixed miso is a mixture of rice, barley, and/or soybean koji. Final miso products associate different colors (red, yellow, or white) and tastes, depending on the proportion of koji. A higher presence of rice or barley koji creates a sweeter taste, while lower ratios of koji produce saltiness. Miso paste is used to prepare soup with seasonal vegetables, seaweed, and seafood [71]. Another Japanese fermented food obtained thanks to *A. oryzae* is hamanatto (sometimes called soy nuggets). It is based on cooked soybeans that are fermented, soaked in brine or in soy sauce, and then dried [72].

Red oncom and black oncom are traditional fermented foods of the West Java cuisine of Indonesia [73]. Red oncom is produced using *N. intermedia* var. *oncomensis* or *Neurospora sitophila* on a mixture of peanut dregs and cassava powder. *R. oligosporus*, *Rhizopus microsporus* var. *oligosporus* and *Mucor* spp. ferment tofu waste to black oncom [57,72,73]. Cheese sandwich paste added with oncom received good ratings in terms of appearance, color, odor, texture/mouthfeel, and saltiness, suggesting that it may be a valuable ingredient [74].

Tempeh or tempe is a traditional Indonesian soybean cake fermented by *R. oligosporus* or *R. oryzae* [57,75]. Besides soybean, other leguminous seeds and cereals are used for tempe preparation [74]. Red kojic rice, referred to as angkak, anka, red qu, Chinese red rice, or *Monascus* fermented rice, is obtained by fermentation of cooked rice with *M. purpureus*, *Monascus anka*, or *Monascus ruber*. It has a specific aroma and purple-red color and is used as a natural colorant in red spirit, red furu, and red rice [76,77]. Gari, a major component of the diet amongst various ethnicities of Nigeria, Benin Republic, Togo, Ghana, Guinea, Cameroon, and Liberia, is a flake made from fermented and roasted cassava using *Aspergillus niger*, *Aspergillus fumigatus*, *Fusarium* spp., *Rhizopus* spp., or *Penicillium* spp. [57]. Furu or tou-fu-ru or sufu is a Chinese cheese-like product with a creamy consistency and easily digestible, made from soybean curd fermented by the mold *Actinomucor elegans* [33].

Moving to the American continent, huitlacoche or cuitlacoche (that means “degenerate corn on the cob”), also called maize mushrooms, Mexican truffles, or maizteca mushrooms, is the smutty maize cob caused by *Ustilago maydis*. This fungal disease is considered an important food source in Mexico and Latin America. Huitlacoche contains 12% protein, oleic and linoleic acids, phosphorus, magnesium, phenolics, and flavonoids [64,78].

Fungi-fermented foods have a typical flavor and enhancements in vitamin, amino acid, protein, and lipid. Food components are enzymatically and chemically broken down during fermentation, and then modified via biotransformation reactions. Fungi fermented foods are more digestible, contain beneficial bioactive compounds produced by the microbes, and help the human organism to create a beneficial gut microbiota. Foods fermented by fungi contain enzymes which could improve the digestion of carbohydrates and proteins. During the fermentative process, fungi act a complex biochemical conversion and produce secondary metabolites, which are not essential for microbial growth. They represent crucial players in the interactions with other organisms, in terms of competition, pathogenesis, and their environment. Many fungal secondary metabolites have beneficial applications, e.g., as antibiotics, antibacterial, antiprotozoals, or medical drugs (immunosuppressant, cholesterol-lowering statins, vitamin A precursor, and indole alkaloid with anticancer properties). Still, others, known as mycotoxins, are harmful to health [46,47,48,53,55,79].

### 3.2. Mycoproteins as Food Ingredients

Compared to dietary proteins of animal origin, mycoproteins can benefit human health as they are characterized by lower energy intake and glycemic response. Furthermore, mycoproteins showed hypocholesterolemic activity and capacity to control body weight [70,79,80]. Different forms of mycoproteins (e.g., dried and rehydrated, canned, frozen) can be used as ingredients in functional foods, such as soups and fortified drinks, biscuits, chicken-, ham- and beef-flavored protein, game pie, and some fish products [66].

Quorn^TM^ (Monde Nissin Corporation, Makati, Philippines) is based on mycoproteins of the soil-associated *F. venenatum* cultured in an open batch system. It is characterized by high amino acids content, low content of fat and calories, and no cholesterol [69,81]. Due to the branched nature of the fungal mycelium resembling the organization of muscle fibers, Quorn has a meat-like texture [56] and is available as burgers, slices, and nuggets [57,64,72,74]. A protein concentrate (46–54% protein content), targeted for vegan consumers, is produced with *A. oryzae* and *N. intermedia* cultured on pea-processing waste [77]. *Pleurotus albidus* myco-proteinaceous flour, compared to conventional chocolate cookies, produces cookies with increased contents of crude protein (about 1.67-fold), dietary fiber (from 1.3- to 4.4-fold based on *P. albidus* myco-proteinaceous flour used), phenolic compounds (about 1.8-fold), hardness, and color [80]. Finally, food byproducts, such as brewers’ spent grain, pasta, and bread scraps can be converted into food products with improved proteins, vitamins (several B vitamins and vitamin D precursors) contents, and dietary fibers after the fermentative process carried out by fungi. For instance, fungi-burgers obtained from stale bread fermented by *N. intermedia* are proposed in Sweden [82]. These fungi-burgers were characterized by higher content of dietary fiber, minerals, and vitamins E and D2 compared to Quorn vegan burger and pork-beef hamburger patties. In addition, their sensory properties were rated as acceptable [82].

### 3.3. Barriers Limiting Consumption of Filamentous Fungi-Based Foods

The development of new food products or foods made with new ingredients must consider healthiness, naturalness, sustainability, dietary behavior, and sensory attributes. In the case of mycoprotein, a notable factor involved is the “mycophobia”, that is the aversion to fungi. More specifically, filamentous fungi are associated with molds and their ability to cause spoilage of foods, to produce mycotoxins, or allergic reactions [57].

Fermented foods such as mold-ripened meat and cheese, Koji and soy sauce, sake, rice-, barley-, soybean- and mixed-miso, hamanatto (also called soy nuggets), red- and black oncom, tempeh, red kojic rice, gari, furu, huitlacoche, and Quorn are associated with different appearances, odors, textures/mouthfeels, and tastes. The meat-like texture of fungal mycelium makes it possible to prepare burgers, slices, and nuggets important in consumer acceptance. At the same time, the sensory attributes are crucial in the acceptance of foods containing proteins of fungal origin. Sometimes, the food based on filamentous fungi may be perceived as difficult to chew and bitter.

The presence of mycoproteins (e.g., dried, rehydrated, canned, frozen) as ingredients in functional or enriched foods could be favorable to texture and consumer preferences. Despite the numerous advantages of fungi consumption, the future of the fungus-derived industry has to deal with the culture of societies. Traditional fermented food is very important in the diet of consumers living in Eastern countries (e.g., China and Southeast Asia), that regularly eat fungus-based foods. Furthermore, Western culture and behaviors have developed mycophobia and disgust with fungal mycelia consumption. Furthermore, the persistent perceptions of harmful fungi contribute to the reluctance to accept fungus-derived foods. Cooperation among academia, government, and industry could alter cultural attitudes and improve the use of fungus-derived proteins as food sources.

Safety concerns could limit the consumption of foods containing proteins of fungal origin. In this case, fungal presence is associated with fungal growth, food spoilage, disease, intoxication or mycotoxins presence, and harmfulness [57,66]. Concerning the safety status of filamentous fungi-based foods, the particular focus must be on controlling the presence of mycotoxins and other harmful substances (e.g., heavy metals). High nucleic acid content is associated with fungi-based foods; indeed, ingestion of purine derived from RNA breakdown increases uric acid concentrations in plasma, which can cause gout and kidney stones in humans [25].

Of course, all foods based on filamentous fungi, especially novel foods, have to be safe for consumption, which sometimes requires a long process. Indeed, Quorn was approved only after 16 years of rigorous testing by the Ministry of Agriculture, Fisheries and Food in the United Kingdom, Switzerland, Norway, USA, Australia, Japan, Thailand, Malaysia, and Canada [54].

## 4. Chances and Issues Related to Microalgae as Sources of Proteins and Other Nutrients

Microalgae are photosynthetic (photoautotrophic), eukaryotic, or prokaryotic microorganisms that can be produced at an industrial scale with low GHG emissions as they convert inorganic carbon, nitrogen, and phosphorus nutrients into biomass [83]. Despite the high biodiversity of microalgae, only a few strains are industrially produced and commercially available. In Europe, most of them belong to *Chlorella* sp., *Nannochloropsis* sp., *Hematococcus pluvialis*, *Spirulina* sp., and *Arthrospira* sp. Other microalgae such as *Tetraselmis* sp., *Tisochrisis lutea*, *Dunalliela salina*, *Phaeodactylum tricornutum*, *Porphyridum* sp., and *Scenedesmus* sp. are also cultured by more than seven companies in Europe.

Microalgal protein concentration greatly varies depending on the species (Table 3). Within a given species, protein concentration is affected by cultivating conditions. When cultivated autotrophically, cell concentration, and hence biomass productivity, is hampered by shadowing phenomena that limit the access of cells to sunlight. However, some species can also be cultivated heterotrophically in fermenters. Heterotrophic culturing of microalgae presents some advantages over autotrophic, such as the possibility to grow on a larger scale, respect for FDA-approved standards and protocols for industrial fermenters, and ability to reach higher cell density since cell shadowing is no longer an issue [84]. The higher growth rates of heterotrophic systems can significantly reduce the time of cultivation, thus improving the economic competitiveness of microalgae production. In addition, when grown heterotrophically, microalgal biomass can present higher protein concentrations compared to their autotrophic cultivation. This is probably due to the reduction in photosynthetic pigments, mainly the nitrogen-rich chlorophyll a, shifting the metabolism to protein production [85]. Xie et al. [86] cultivated *Chlorella vulgaris* with a nitrate concentration shift under heterotrophic conditions for protein enhancement. Under optimized conditions (0.18 g L^−1^ nitrogen, in 38 h), 2.45 g L^−1^ of biomass were obtained with 44.3% of proteins [86]. An increase in biomass (14 g L^−1^) and protein content (60.1%) may also be obtained upon sequential heterotrophic/autotrophic cultivation of *Chlorella* [87].

Most microalgae have all the essential amino acids and fulfil the FAO/WHO values standard for essential amino acids for children aged 2–5 years (Table 4). Their amino acid composition is comparable to soybean, a conventional vegetable source of dietary proteins. Their protein profile is affected by diverse and harsh environmental stimulation such as nitrogen increase in the cultivation medium.

In addition to being considered as sources of dietary proteins, microalgae are also rich in omega-3 and omega-6 fatty acids, with potential anticancer, immunomodulatory, and cardiovascular disease-preventing activities [89] (Supplementary Table S2).

### 4.1. Use of Microalgae as Additional Food Ingredients

Microalgal biomass produced under heterotrophic growth can be directly consumed as a food supplement or as an ingredient in food products, with the aim to increase protein content and health-promoting properties. Coporgno et al. [98] produced a meat analogue using high moisture extrusion-based with yellow, heterotrophically cultivated *Auxenochlorella protothecoides* combined with soy concentrate protein extrudates. The best mechanical properties were obtained with 30% microalgae incorporation at a moisture level of 60% [98].

Few fermented products are reported using microalgae as an ingredient. Scieszka et al. [99] developed a soya drink supplemented with *C. vulgaris* and fermented with the probiotic strain *Levilactobacillus brevis* ŁOCK 0944. The soya drink and *C. vulgaris* were sufficient sources of nutrients for *L. brevis* ŁOCK 0944 and increased the survival of the lactic acid bacteria in the drink. Kemiri et al. [100] produced a gluten-free bread using *Nannochloropsis gaditana* L2 as an ingredient at a 3% ratio. They observed an increase in protein content and higher content of iron and calcium, compared to the unfortified bread, together with a balanced profile of fatty acids. The product with 3% of *N. gaditana* had remarkable sensorial results, compared to the control bread, presenting the highest score in terms of global appreciation. Barkalla et al. [101] studied the effect of *Arthrospira platensis* (spirulina) fortification on the fermentation process, texture, nutraceutical, and sensory characteristics of yogurt. The authors found that the addition of 0.25% of *A. platensis* accelerated fermentation. In addition, it was hypothesized that the high contents in dietary fibers and proteins of the microalgal biomass played the role of physical stabilizers improving the mouthfeel and enhancing syneresis and apparent viscosity. The application of *A. platensis* biomass (0.5–1%) on feta-type cheese acidified with probiotic *Lacticaseibacillus casei* significantly increased the number of viable counts of probiotic bacteria after 60 days compared to control samples [102]. Feta cheese produced with *A. platensis* exhibited a softer texture which led to an easier disintegration and chewing. No significant differences were observed between the control and cheese containing microalgae [102]. Thirumdas et al. [103] developed fermented Spanish “chorizo” sausages with 3% of *Chlorella* or *Spirulina* and evaluated the impact of biomass addition on physicochemical and nutritional properties. The protein content of sausages with *Spirulina* and *Chlorella* reached 34.89% and 34.66%, respectively. Hardness, adhesiveness, and gumminess of sausages were increased in the presence of algal proteins and the produced sausages showed a dark green color decreasing the redness of meat. The sausages with *Chlorella* addition also showed a higher ratio of essential to non-essential amino acids than *Spirulina* sausage.

### 4.2. Sensory and Nutritional Issues Related to the Dietary Intake of Microalgae

**Figure 1:**
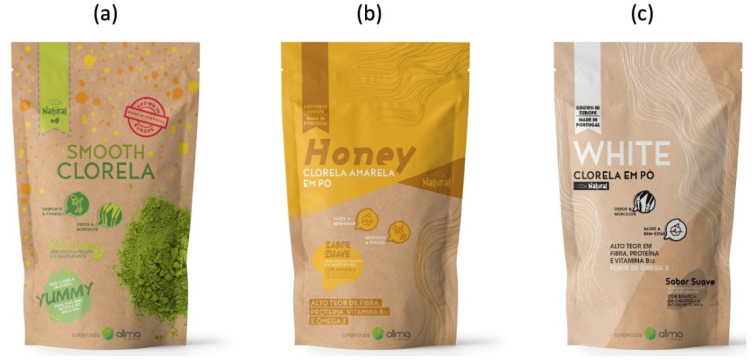
Commercial *Chlorella* produced by the Portuguese company Allmicroalgae: (**a**) lime, (**b**) yellow, and (**c**) white. Source: https://www.allmashop.com/pt-pt/ (accessed on 11 July 2022).

Color, taste, and odor of microalgae can be a bottleneck for the development of microalgal-supplemented foods limiting the ratio of biomass incorporation in the formulation [89]. The degree of acceptability by consumers depends on the traditional diet of the population; for Western populations, microalgae are still not a common food ingredient [104]. However, the sensory characteristics of microalgae can be modulated through heterotrophic cultivation and mutagenesis. Heterotrophic cultivation substantially decreases the chlorophyll content of the microalgal biomass, reducing or eliminating its greenish color and affecting also the taste and odor [89]. A Portuguese company, Allmicroalgae, produces a “Smooth *Chlorella* Powder” characterized by lighter green color and smoother flavor (Figure 1a). This company also produces “Honey”, a yellow *Chlorella* (Figure 1b) and “White” a white *Chlorella* (Figure 1c). Microalgal mutants with more acceptable colors can be obtained upon the application of random mutagenesis followed by strain selection. Using this strategy, Schüler et al. [85] produced *C. vulgaris* in three colors: yellow, white, and lime. The authors also observed that the chlorophyll concentration in the biomass was inversely proportional to the protein content as the white strain showed the highest protein content, reaching 48.7%, which meant a 60% increase as compared to the wild type.

An important factor to consider in the application of microalgae biomass for food is the digestibility, as the robust cell wall of certain strains can restrict the access of the digestive enzymes to the cell components [105]. Niccolai et al. [106] investigated the digestibility of 12 microalgae using pepsin and pancreatin as enzymes. The authors found the highest digestibility for cyanobacteria, mainly *A. platensis* F&E-C256 (78% dry matter, 86% organic matter, 79% carbohydrate, and 82% crude protein digestibility), *Chlorella sorokiniana* F&E-M-M49 and *C. vulgaris* Allma. Marine species, such as *Tetraselmis suecica*, *Phaeodactylum tricornutum*, *Nannochloropsis* sp., and *Porphyridium purpureum*, were the least digestible [106]. This is probably due to different cell wall structures. *Nannochloropsis* has a thick cell wall composed of cellulose and algaenans that may reduce digestibility. *Porphyridium* cells are covered by polysaccharides that can form stable complexes with proteins and reduce cell access to proteolytic enzymes. On the other hand, green algae such as *T. suecica* have a cell wall composed of cellulose, hemicellulose, pectic compounds, and glycoproteins that can interfere with the action of digestive enzymes. Additional treatments on microalgal biomass could be performed for increasing protein digestibility.

In addition to showing low protein digestibility, microalgae could have lower content of some vitamins (e.g., B12 and D3) [107,108] and minerals (e.g., iron) than meat and dairy products [109]. However, vitamin D3 can be produced by microalgae (e.g., *Dunaliella salina*, *Nannochloropisis oceanica*, and *Nannochloropisis limnetica*) upon UV-B exposure [110]. Elderman et al. [111] studied the composition in the vitamins B2 (riboflavin), B3 (niacin), B9 (folate), and B12 (cobalamin) in microalgal biomass and found interesting contents of all four in *Chlorella* sp. (B2 33.6 μg/g; B3 0.32 mg/g; B9 25.9 μg/g; B12 2.4 μg/g), *Arthrospira* sp. (B2 40.9 μg/g;B3 0.22 mg/g, B9 4.7 μg/g; B12 2.4 μg/g), and *N. gaditana* (B2 22.1 μg/g; B3 0.11 mg/g, B9 20.8 μg/g, B12 0.25 μg/g) powders. Concerning minerals, Santhakumaran et al. [112] studied the mineral composition of 25 microalgae species. They found that they are all rich sources of one or another kind of minerals or trace elements. For example, *Bracteacoccus minor* was identified as a good source of iron (10.2 mg/g) and *Chlorococcum humicula* as a good source of zinc (1.1 mg/g) and cobalt (0.05 mg/g) [112].

### 4.3. Legislation and Additional Issues for Future Research

In Europe, microalgae production is still limited by a series of technological, regulatory, and market-related barriers [91]. The European legislation is one of the main barriers to using microalgae as a novel protein source. Microalgae are considered a novel food and new species must be submitted to a novel food application before being marketed. A novel food is defined as a food that has not been consumed to a significant degree by humans in the EU prior to 15 May 1997, when the first Regulation (Regulation EC No 258/97) on novel foods came into force. Up to date, 11 macroalgal and nine microalgal species have been authorized as foods or food ingredients and listed as “not novel” (or old) in the EU Novel Food Catalogue. The microalgae used prior to May 1997 in Europe and thus authorized as food in the EU are *Aphanizomenon flos-aquae* from Klamath Lake, *A. platensis*, *Chlorella luteoviridis*, *Chlorella pyrenoidosa*, and *C. vulgaris* (European Union, Novel Food catalogue). The diatom *Odontella aurita* was authorized in 2005 (European Union, 2005), and, in 2009, docosahexaenoic acid-rich oil from *Ulkenia* sp. was approved as a novel food ingredient (European Union, 2009). In 2014, *Tetraselmis chui* and astaxanthin from *Haematococcus pluvialis* were also approved as novel food ingredients [97].

Future research on microalgae as sources of dietary proteins should focus on the culture conditions allowing to improve their protein content and amino acids profile. For example, strategies involving concentration shifts of nitrates have been devised to improve the protein content of heterotrophically produced *Chlorella vulgaris*, increasing by 44.3% of the protein content. Although the amino acid profile was not altered by the changes in cultivation method imposed, it was already balanced, which is a common feature in microalgae [86]. Like many vegetable sources, microalgae present low recovery rates of protein, mainly due to the presence of thick cell walls. Hence, the application of cell lysis is vital to increase the digestibility of microalgae. Different cost-effective and scalable alternatives exist including chemical (e.g., treatment with solvents or alkali solutions), physical-mechanical (e.g., ultrasonication, grinding), or enzymatic [113]. Another topic that is poorly explored is the allergenicity of microalgal proteins. Novel protein sources have not been on the markets long enough for allergies to be detected or to establish proteins responsible for allergenicity. So far, only a few studies report allergic reactions to microalgae, such as an anaphylactic reaction to the b-chain of phycocyanin C of *A. platensis* and acute tubulointerstitial nephritis developed after ingestion of *Chlorella* tablets [114]. Recently, a study reported the occurrence of proteins with significant sequence homology to those of known allergens (like those occurring in fish and shellfish) [115]. Although clinical studies remain to be performed, this study indicates that microalgae might be potential allergens. As it is probable that some level of processing needs to be done to microalgal biomass to increase its value as a protein source, it remains to be seen how it reflects on the allergenic potential of this biomass.

## 5. Pros and Cons of Vegetables as Sources of Dietary Proteins

Various vegetables have been proposed as sources of dietary proteins: legumes (soybean, pea, bean, chickpea, lupin, fava bean, cowpea), cereals (rice, wheat, millet, sorghum, maize, and barley), and pseudocereals (amaranth, quinoa, and buckwheat) [116]. This section will not treat the use of vegetable matrices as ingredients for meat analogues. The reader may find more information about that in a recent review [117].

With some differences among different crops, legumes contain approximately 21–25% of protein, 1–1.5% lipids, 60–65% carbohydrates, and 2.5–4% ash [118]. Some of them, such as soybean [119] and lupin [120], have been reported as having a higher content of protein (Table 5). Among plant sources and particularly legumes, soybean is the most used. Soybean may be the base ingredient for many foods including cheese, drinks, miso, tempeh, tofu, salami, and vegetarian meat substitutes [96]. Although soybean proteins are characterized by amino acid composition and essential amino acid content very close to animal requirements, they are deficient in the essential amino acid methionine, involved in several health-beneficial reactions [121]. A suboptimal concentration of methionine limits the nutritional value of soybean and therefore previous studies aimed to increase this amino acid through dietary supplementation [122,123,124].

The protein content in cereal grains is relatively lower compared to legumes seeds. It ranges from 7 to 17% [126], with the majority coming from storage proteins [129]. Nevertheless, cereal grains provide over 200 million tons of protein for the nutrition of humans and livestock, which is about three times the amount derived from legumes [116]. In addition to considerable high amounts of proteins and carbohydrates, cereals, such as oats and barley, contain a wide range of phenolic compounds with antioxidant activity. Oat is well accepted by consumers [129]. It contains high amounts of valuable nutrients such as soluble fibers, unsaturated fatty acids, vitamins, minerals, and phytochemicals [130], high protein content (ranging from 10 to 18% ca. depending on variety), and a good amino acid balance [131]. Oat and barley are also sources of ß-glucans that have been shown to have numerous health benefits, such as a reduction of cholesterol and glycemic response, modulation of gut microbiota, management of hypertension, and reduction in the incidence of metabolic syndrome [130].

Protein content of pseudocereals, such as amaranth, buckwheat, and quinoa, varies in the range 12.5–16.5% [125] and the concentrations of essential amino acids, particularly cysteine and methionine, are higher than in common cereals such as rice and maize [132].

Cereals are a good source of methionine and cysteine, two essential amino acids, and B-complex vitamins, but are scarce in lysine. On the other hand, most legumes are rich in lysine but low in sulfur-containing amino acids (Table 5). Thus, the composition of cereals and legumes results in a good complementarity of a number of nutrients [133]. It has been shown that for each portion to provide equal parts of protein mass, the optimal ratio of cereals and legumes is 70:30 [133].

Altogether, plant-based diets have been positively associated with a healthy lifestyle. Legumes might reduce the risk of suffering cardiovascular disease [134], metabolic syndrome, and type 2 diabetes, while they provide substantial benefits in terms of weight control and gastrointestinal health [135]. Healthy benefits of the proteins of such vegetable matrices also derive from the release of bioactive peptides with antimicrobial, antihypertensive, hypocholesterolemic, immunomodulatory, antioxidant, antithrombotic, and antitumor effects [136].

Plant-based proteins also exhibit functional properties that make them suitable for food formulation, gluten free or protein-enriched products, or bio-fortified cereal-based products [137]. These properties include water holding and oil binding capacity, bulk density, gelation ability, foaming capacity, and emulsifying activity. These properties depend on protein and peptide structure and on the interaction with carbohydrates, lipids, other proteins, or water [137].

However, the use of these vegetable matrices presents some limiting aspects. Plant proteins can be responsible for allergies [138], celiac disease [139], and phytoestrogens intake [137]. Legumes and cereals may also contain anti-nutritional factors (ANFs), which can interfere with the absorption of many nutrients and thus reduce their bio-accessibility [140]. ANFs include lectins, protease inhibitors, phytic acid, phenolic compounds (tannins and saponins), α-galactosides, and alkaloids [141]. Lectins and protease inhibitors are proteinaceous compounds responsible for sugar-binding activity and decreasing protein digestibility, respectively. Protease (such as trypsin and chymotrypsin) inhibitors are present mainly in the seeds of soybean (20 g/kg), white beans (3.6 g/kg), and chickpeas (1.5 g/kg) [140]. Phytic acid is found in most cereals, nuts, and legumes and strongly binds minerals such as iron, zinc, calcium, and magnesium. It can also form complexes with protein and digestive enzymes, lowering protein solubility [140]. Alkaloids are secondary metabolites found in Leguminosae (mainly in lupin) that are responsible for a bitter taste that makes them unappetizing for humans and animals, and toxic for the organism itself [140]. Tannins have astringent properties and can bind salivary glycoproteins, reducing in palatability and bitter taste. They also decrease protein digestibility as well as amino acid availability. Legumes also contain variable concentrations of α-galactosides of sucrose (raffinose, stachyose and verbascose), fermented by intestinal microbes causing abdominal pain, diarrhea, and flatulence [140]. Finally, protein-rich raw materials can be a source of biogenic amines (BAs), such as histamine, tyramine, tryptamine, putrescine, cadaverine, and phenylethylamine, low molecular weight nitrogenous compound, coming from the decarboxylation of amino acids performed by bacteria, molds, and yeasts naturally present in such matrices [142]. Some BAs (e.g., histamine, tyramine) may be responsible for toxic effects in consumers, resulting in several symptoms such as nausea, headache, palpitations, or oral burning. Presence of ANFs in cereals and legumes may be counteracted by applying traditional techniques such as soaking, fermentation, cooking, roasting, or germination before consumption [143].

### Tradition-Based Innovation in Fermented Foods of Vegetable Origin

Fermented foods and drinks have always played a fundamental role in human nutrition and they differ depending on cultures and geographic regions [144]. In general, world dietary habits can be distinguished based on the predominant type of cereal-based foods consumed, often after fermentation: (a) in East Asia, the diet is mainly rice-based; (b) in Western Asia, Europe, North America, and Australia, we can mainly find bread made from wheat or barley; and (c) porridges made from sorghum or corn in Africa and South America, and cassava and root/tuber-based staple foods are also widespread in Africa [145]. In the Indian subcontinent, cereals and legumes are fermented in large quantities, often together, as in the production of dosa, idli, adai, vada, and pupadum. In East and Southeast Asia, legumes (along with fish) are the most important fermented foods. In these regions, cereal products may also be co-fermented with legumes, as in the case of miso (rice, barley, or other cereals with soybeans) and soy sauce (wheat and soybeans) [145]. Although among legumes soybean is the predominant substrate for fermentation, other legumes, such as chickpea or pigeon pea, could also be used [146]. For example, the chickpea sourdough also known as “sweet yeast” or “chickpea yeast” is a well-known traditional method used in various Mediterranean and Balkan countries, as a leavening agent that confers a distinctive flavor and taste to food [147].

**Figure 2:**
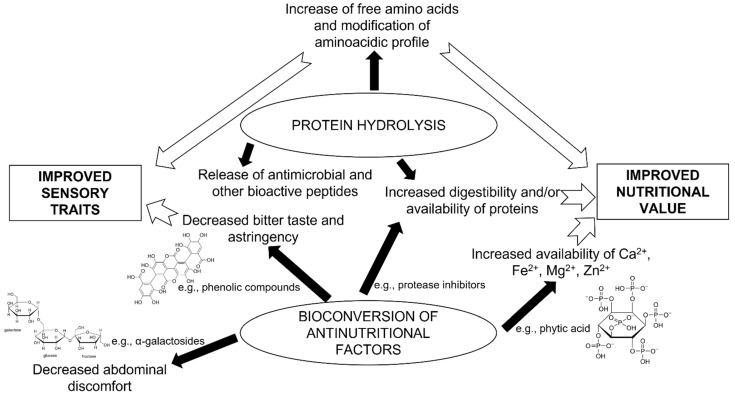
Schematic representation of some mechanisms occurring during fermentation of cereal, pseudocereal, and legume flours and benefitting food quality aspects.

Among the microorganisms used in food fermentation, we can mention bacteria (e.g., *Lactobacillus* spp., *Lactiplantibacillus* spp., *Levilactobacillus* spp., *Streptococcus* spp., and *Bifidobacterium* spp.), filamentous fungi (e.g., *Aspergillus* spp., *Mucor* spp., and *Rhizopus* spp.), and yeasts (e.g., *S. cerevisiae*) [144]. Lactic acid fermentation is a natural way of increasing concentrations of vitamins and essential amino acids, decreasing ANFs, and improving food appearance, flavor, and aroma (Figure 2). Lactic acid bacteria (LAB) are the main, if not the only, actors of this fermentation, which, from a biochemical point of view, solely consists of conversion of carbohydrates (generally mono- or di-saccharides) into organic acids (lactic acid and, sometimes, acetic acid), carbon dioxide, and ethanol [144]. However, during LAB-driven fermentation, other biochemical processes occur, such as protein hydrolysis and release of volatile organic compounds and antifungal metabolites. Application of lactic acid fermentation to food considerably modifies its texture and sensory properties, extends shelf-life, and counteracts spoiling and/or pathogenic microorganisms [144].

In recent years, researchers and producers have been moving more and more towards the formulation of novel foods legumes, pseudocereals, and minor cereals through fermentation [147,148,149,150,151,152] to increase the nutritional value of conventional food, such as bread and pasta [153]. Montemurro et al. [154] investigated the nutritional and functional aspects of wheat, barley, lentil, chickpea, and quinoa seeds by combining both germination and fermentation with a selected pool of LAB. Experimental breads fortified with germinated flours sourdoughs were characterized by an increased release of peptides and free amino acids, phenolic compounds, and soluble fibers, and intense decrease of several ANFs, compared to traditional wheat flour bread. In addition, they were characterized by peculiar sensory profiles and showed higher protein digestibility and lower starch availability.

Microbial metabolism in vegetable-based substrates increases proteins solubility and availability because some metabolic pathways hydrolyze ANFs (some of which decrease protein availability) in those substrates [144]. For example, *Bifidobacterium* significantly increased the protein content of soybean-based drinks [155]. In addition, many microorganisms cause variations in the amino acid profiles. For instance, fermentation of soybean with *Lactiplantibacillus plantarum* resulted in increased essential amino acids such as lysine [156]. This occurs for LAB, such as *L. plantarum*, because their growth in all substrates, including the vegetable-based ones, depends on a complex proteolytic system, which generates FAA from proteins and peptides [157]. Although most studies have focused on mono-culture fermentation, the use of two or more microbial strains, if they are compatible, could further improve the amino acid profiles of vegetable-based foods [144]. For instance, compared to peanut fermented with monocultures of *Lactobacillus acidophilus* and *L. plantarum*, concentrations of proteins and some essential amino acids (lysine, methionine, and tryptophan) increased when a mixed starter, composed of the two lactobacilli, fermented this matrix [158]. Indeed, it is well known that one single microorganism usually does not harbor the whole enzyme portfolio for an almost complete protein hydrolysis. Therefore, if properly selected, two or more microbial species (or, sometimes, even strains) cooperate to get a greater degree of protein hydrolysis than what is achievable by each single species. This may also occur when combining LAB with filamentous fungi. For instance, when whole-grain oat was co-fermented with *L. plantarum* and *R. oryzae*, lactobacilli grew much better than in monoculture, possibly because fungi degraded polymers to simple molecules that stimulated the growth of lactobacilli. In addition, the co-fermentation product was richer in soluble proteins and small peptides (possibly including angiotensin-converting enzyme inhibitory peptides) [148].

Co-culture of *L. plantarum* DSM33326 and *L. brevis* DSM33325 was used by Pontonio et al. [159] to ferment a blend of rice, chickpea, and lentil flours to obtain a novel yogurt-style snack. The fermentation led to an improvement of the nutritional profile of the matrix. Total FAA were ca. 67% higher in the fermented product, with respect to the unfermented control due to the proteolysis operated by the selected starters on the native proteins. Proteolysis occurring during fermentation also increased the level of protein digestibility, resulting in ca. 18% higher than the unfermented matrix.

Selection of an appropriate starter culture is challenging because some parameters have to be considered such as the starting matrix, the stressful conditions of the fermentation process, the metabolic activity of the strain, and the desired result [160]. For example, based on a selection process among 70 strains of LAB according to pro-technological and functional features, Pontonio et al. [161] selected *L. plantarum* T6B10 and *Weissella confusa* BAN8 as a mixed starter culture to obtain high nutritional wheat bread fortified with hull-less barley and emmer brans. Compared to the control, represented by wheat-based bread, fortified bread had high levels of proteins (up to ca. 13% of dry matter) and higher protein digestibility (ca. 40%), thus hypothesizing that proteolysis by LAB played a key role.

## 6. Advantages from “Hybridization”: The Case of Vegetable/Milk Mixed Foods

Cereal-, pesudocereal-, and legume-based fermented food items represent one option to increase vegetables consumption, thereby allowing to partially replace meat as dietary source of proteins. However, these food items, especially when containing legumes, are characterized by lower protein digestibility and content (e.g., leucine) or deficiency (e.g., sulfur amino acids, lysine) of some essential amino acids, compared to animal proteins [162]. Therefore, consumption of mixed fermented products (MFPs), made of milk and vegetable blends, could be one possible solution for such a nutritional issue [163]. For example, methionine would be more available from animal/pea protein mixes than from pea protein alone, since pea protein is poor in this essential amino acid [163]. Another advantage of such blends, with respect to food solely based on vegetables, is to increase acceptability, through masking or reducing the green and beany notes given by legumes, and keeping some “familiar” sensory attributes, such as those of dairy products. Finally, compared to food solely based on milk, MFPs have a lower environmental footprint. Therefore, the development of mixed vegetable/animal fermented food represents an area of potential innovation for providing sources of dietary proteins meeting consumers’ expectations.

MFPs are traditionally consumed in many areas of the Middle East, Africa, and Asia [164]. The milk part is generally composed of whole milk (cow, sheep, goat) or buttermilk, whereas the vegetable part is composed of cereals and/or legumes. Fermentation plays a major role in providing food with the typical sensory traits, increasing the nutritional value (e.g., through reducing the amount of ANFs) and increasing the healthy features (e.g., through release of bioactive compounds, such as immunomodulating peptides) [165].

### 6.1. Traditional MFPs

MFPs are quite popular foods in Balkan, Mediterranean, and Middle Eastern countries and represent important dietary sources of proteins, lipids, and carbohydrates. They are also a source of minerals, whose qualitative-quantitative profile reflects the vegetable used [166,167]. Their manufacture differs from country to country and even within the same country, but the main ingredients are yogurt and wheat flour or parboiled wheat (bulgur). Although they may be purchased from retailers, most MFPs are homemade and their composition considerably varies depending on the ingredients.

Among these products, the most studied is tarhana, a traditional Turkish fermented food. It is prepared by mixing wheat flour and yogurt (typically in the ratio of 2:1) and adding yeast, salt, spices, and a variety of cooked vegetables (e.g., tomatoes, onions, green pepper). The mixture is fermented at 25–30 °C for one to seven days [168] and finally dried at room or controlled temperature to reduce the moisture content to about 10%, thus extending shelf-life [169]. Dried MFPs could be consumed mainly in three different forms: (a) as a powder added to soup preparations; (b) with bread, after rehydration in a small quantity of water, obtaining a porridge-like or soft cheese-like product; or (c) as an ingredient in various recipes. Grounded dry tarhana is traditionally used to make soups that are consumed at breakfast or supper, alone or with vegetables and oil. It has a sour taste and yeasty flavor but may assume several other flavors depending on the vegetables and spices used as additional ingredients. Similar to tarhana, kishk, traditionally consumed in Lebanon, Siria, Palestine, Jordan, and Egypt, is usually reconstituted with water and served as a hot gruel, in which other ingredients (e.g., vegetables, spices, garlic, herbs, dates) could be added to form the base of savory or sweet dishes [164].

Analyses of tarhana samples from different regions of Turkey showed on average 10.2% moisture, 16.0% proteins, 60.9% carbohydrates, 5.4% fat, 1.0% fiber, 3.8% salt, and 6.2% ashes [169]. Similar average values were found in trahanas, a similar product made in Greece [170]. Kishk contains proteins (18–22%), fat (4–11%), and carbohydrates (30–70%) [166,167]. Culture-dependent analysis of tarhana revealed LAB as dominant microorganisms. *Pediococcus acidilactici* (27%), *Streptococcus thermophilus* and *Limosilactobacillus fermentum* (formerly *Lactobacillus fermentum*) (19%), *Enterococcus faecium* (12%), *Pediococcus pentosaceus* (7%), and *Leuconostoc pseudomesenteroides* (5%) were the most represented LAB species. Although production sites had peculiar LAB profiles, *P. acidilactici* and *S. thermophilus*, originating mainly from yogurt, were the most frequently encountered species [171]. Another study showed that *L. plantarum* was the main species in homemade tarhana, whereas *L. brevis* and *Companilactobacillus alimentarius* (formerly *Lactobacillus alimentarius*) were dominant in commercial tarhana [172]. 16S metagenetic analysis performed on tarhana along the fermentation, showed high bacterial diversity at the beginning of the process, with *Lactobacillus*, *Bacillus*, *Enterococcus*, and *Streptococcus* as dominant genera. *Clostridium* and *Bacillus* became dominant at the end, but *Clostridium* was not detected after drying. The final product harbored *Bacillus* and *Streptococcus* [173]. The fungal community of homemade and commercial tarhanas shared the presence of the yeast species *Pichia kudriavzevii*. Homemade tarhana also harbored *S. cerevisiae* and *Kluyveromyces marxianus*, whereas commercial tarhana also harbored *Candida humilis* (syn: *Kazachstania humilis*) and *Candida glabrata* [174]. The microbial community of tarhana is affected by the ingredients used, as shown for cornelian cherry tarhana, which harbored, in addition to *P. kudriavzevii* (indicated as *Candida krusei*) (11%), *Hanseniaspora uvarum* (32%), *S. cerevisiae* (20%), *Torulaspora delbrueckii* (19%), and *Clavispora lusitaniae* (indicated as *Candida lusitaniae*) (9.3%). *Limosilactobacillus reuteri* (formerly *Lactobacillus reuteri*) and *Enterococcus* spp. completed the picture of the microbial community [175].

### 6.2. Novel MFPs

Innovation in MFPs may consist in: (i) replacement of the type of flour and ingredients; or (ii) use of selected microbial strains and/or unprecedented combinations of ingredients. Novel tarhana may be produced by substituting, partially or totally, wheat flour with flour from other cereals, pseudocereals, and/or leguminous seeds [176]. Because MFPs contain phytic acid, an ANF reducing mineral availability and digestibility [177], such replacement could impact on nutritional features of MFPs. During fermentation of wholemeal wheat kishk, mineral availability increased compared to wholemeal bulgur kishk, as shown by its lower content of phytic acid and higher amounts of Ca, Fe, Mg, and Zn [177]. Increase in mineral contents, along with constant or improved sensory traits of novel tarhana, was obtained through partial replacement (up to 40%) of wheat flour with buckwheat flour [176]. On the other hand, partial substitution (5%, 10%, and 15%) of wheat flour with rice bran led to tarhana with higher content of phytic acid, proteins, and antioxidants. It lowered sensory scores more than traditional tarhana [178]. Partial replacement of wheat flour with corn bran improved sensory scores compared to traditional tarhana, although with lower protein content [178]. A further option to increase the mineral availability of tarhana is to use different phytase sources (baker’s yeast, barley malt flour, microbial phytase) that decrease phytic acid content [179].

Selection of microbial strains allows to control fermentation of vegetable/milk mixed foods, thus standardizing the process and avoiding the occurrence of undesired bacterial genera (e.g., *Bacillus*, *Clostridium*). In some studies, microbial consortia were empirically selected [180,181,182], whereas other researchers carefully designed the fermentation agents [44,183]. Three LAB (*Lactobacillus farciminis* PFC83, *L. casei* PFC90, *C. alimentarius* PFC91) and two yeasts (*P. kudriavzevii* PFC12, *C. humilis* PFC138), isolated from traditional tarhana, were used (singly or in combination) to control tarhana fermentation better. Esters and alcohols accumulated during fermentation in different proportions that depended on the microbial association used as starter. Lactic, succinic, and acetic acids were the most prevalent organic acids. *C. alimentarius* and *P. kudriavzevii* were correlated with the accumulation of esters and organic acids and the tarhana obtained using these microorganisms received the highest sensory scores [184]. The appropriate selection of strains has been successfully used to reduce off-flavor (e.g., green, pea notes) in milk-legumes mixes [44,185]. Such an approach could be applied to conceive other novel MFPs with well-balanced sensory and nutritional properties. Compared to pea gels, fermented pea gel-milk mixes were characterized by notes of dairy products, with almost no characteristic legume notes (e.g., pea, wood, dried fruit, grass) [44]. Use of plant protein additives was scouted in fermented skim milk, improving the physico-chemical and sensory properties and increasing the nutritional value [186].

Gluten-free tarhanas, produced by substituting wheat flour by red, green, or yellow lentil flour, showed significantly increased amount of crude protein (26–30%), total dietary fiber (12–19%), mineral concentrations, and antioxidant activities compared to the traditional, wheat flour-based tarhana [187]. Similarly, gluten-free tarhana containing various cereals (rice, corn, buckwheat) and legumes (bean, chickpea, lentil) proved to be a protein-rich food, with high mineral content, fatty acids, and with high digestibility and anti-oxidative activity [188].

## 7. Insects as Sources of Dietary Proteins

Insects have been used as a food source by humans from the prehistoric very early times of our rise [189]. There was a general prohibition for insect-eating in early historical times, with valuable allowance for locusts in otherwise forbidding cultures [190]. The appeal of insects as alternative food sources originates from concerns for nourishing the world population in the near future [191,192], the need for new food sources mitigating food shortage [193,194], and the need to mitigate microelements dietary deficiencies [195]. Different insect species could be used, as such or as an ingredient, as a source of dietary proteins [196,197,198,199]. Despite most of information about insects as food remaining anecdotic [200,201], several proteins from insects originate from environments safer than traditional collection techniques, as in the case of silkworm [202,203,204], honeybees brood [205], and mealworm [206].

### 7.1. Application of Insects to Food Products

Several biscuits [207,208,209], snacks [210], enriched cornflour [211], different breads [212,213,214,215,216], meat batter [217], and sausages [218] can be prepared using insects. Cricket powder [219] may supplement wheat-based pasta. These food items are characterized by higher protein content and enhanced protein quality than their conventional counterparts [206,220,221,222,223,224,225]. Several studies considered acceptance of cookies, honey spread, extruded rice products, crackers, and similar preparations with insects (whole or their parts) directly perceivable [210,226,227,228,229,230,231,232]. Although almost totally unexplored, food insects often show good taste and flavors similar to walnuts, hazelnuts, almonds, or shrimps and crustaceans.

Fermentation has a vast and diffused impact on the food-oriented treatment of insects. First, insects themselves carry and disperse a guild of appropriate microorganisms helping them to establish an insect-favorable habitat. Phytophagous [233] and sarcosaphrophagous *Diptera* [234] often use their bacteria to digest their feed. Mouthparts and gross maggot morphology are specialized purposely for the job, even in the less specialized *Hermetia* [235]. Microorganisms thrive in insects’ guts (i.e., food channels) [236] and insect breeding and rearing environments [237]. Another influx of insect-driven fermentations consists in their ability to modify the microbiome of the host organisms [238] and, thus, in vitro fermentation model [239,240]. Generally, biasing or modification of fermentations originates from changes in diet components. Moreover, the insect body or carcasses possess a peculiar microbiota that can be a starter in bread enriched with cricket powder [241]. Fermentation of insect paste [242] or seasoning sauces may enhance food palatability and acceptance. Bacteria have a different exciting role in recycling byproducts associated with insect rearing [237]. In captive mass breeding of well-accepted Palm Weevil grubs [233], bacteria play a relevant part in pre-digesting the artificial diet matter. A further argument about food insect association with microorganisms consists in fermenting bacteria associated with processed, stored, and traded food insects [243].

### 7.2. Issues and Solutions Related to the Use of Insects as Protein-Rich Foods

The inclusion of edible insects as food ingredients is mainly intended to enrich the protein content. However, the concentration of proteins in insects is overestimated due to high non-protein nitrogen content. A further issue to be faced by researchers and manufacturers is the meaning of “ash” content in insects [195,228,229]. Indeed, while ash from plant material consists of minor degradable food components (e.g., lignin or lignocellulosic matter), the less digestible part of insects consists of the cuticle that, notoriously, passes through the vertebrate gut almost untouched [230]. The cuticle itself can enter nitrogen-rich food matter, and the definition of ash shall be possibly reassessed for the purpose.

Given that insects’ consumption is not familiar among Western consumers [231,232,233,234,235], future research should be focused on increasing acceptance of edible insects. Another issue is insects’ food safety, whether collected in the wild or in cultivated fields. The direct or cross (crustaceans) reactivity to insect allergens seems related to the insect species [236,237], but the treatment of insect proteins may significantly reduce [238] the impact of the phenomenon. As well as allergens, insects may be contaminated by chemical or biological agents. Insects with a pluriannual lifecycle, often feed on soil rich in organic matter or wood, or slowly elaborate degraded plant or organic matter host bacteria possibly associated with protozoa. In many cases, the insect gut specializes in “caeca” or other slow-speed adaptations to allow the action of symbiotic microorganisms. Specializations include termite or scarab larvae proposed as tribal food but thriving on plant decaying matter in litter or soil. Local mini-livestock insects’ use for food [239,240] seems proper only in the case of local and tribal use because of the low safety of a food directly gathered from the wild. Storage, stabilization, and conservation of insect powders [229], paste, or whole insects represent the main links of the insect-based food chains, which, of course, must apply the HACCP approach [241]. From this point of view, the research could focus on some steps in the post-embryonic development of the insects that are easier to manage and process. In detail, the pupa of holometabolous insects is self-cleaned and microbiologically safe, offering a phase of development to choose for postponed or diverse transformation. It is worth mentioning that the pupal stage can be induced and sustained alive by temperature control in storage facilities for other use in transformation. Evidence from the insect bionomic study suggests the case by correctly choosing the experiment frame to obtain results that will easily move to a profitable scale of applications.

Finally, a wild-obtained food resource can be accepted only if the resource can sustain the user’s food intake. Insects’ diet needs an appropriate assessment because they influence insect-associated microorganisms, control the amount of water available for the insect, and may affect the nutritional value of food. A particular focus should be dedicated to using substrates for insect rearing that are different from food sources (e.g., wheat) commonly used by humans (e.g., wheat) because the insect feed can end in the food.

## 8. Implications and Limitations

This review could inspire food producers, especially start-up enterprises, to ideate novel products and/or processes through combination of fermentation technologies and meat-alternative protein sources. Indeed, fermentation with selected, or even engineered microorganisms, would allow to improve sensory attributes, and to increase the nutritional value of meat-alternative food items. In addition, this review fosters positive contamination among researchers with different backgrounds, e.g., microbiology, biotechnology, agronomy, human nutrition, and entomology.

Nevertheless, we should highlight some limitations of this review. First, it does not treat seafood and culture meat, representing other alternative protein sources. Current demand for seafood far surpasses the sustainable capability of sea, which boosted aquaculture [21]. However, even aquaculture poses some sustainability and animal welfare issues. Cultured meat, in addition to still not being fully framed in regulatory assets, shows uncertain or low sustainability gains, because it requires much processing [244]. Further issues related to culture meat are: (i) unlike from algae and insects, humans have never consumed it, and (ii) the production of broth to culture meat may require more resources (including some ingredients of animal origin) than those needed for meat production [245]. Another intrinsic limitation of this review is that, because of limited available space, it is far from being exhaustive and does not treat in detail some aspects, such as consumers’ acceptability and gray zones of safety and of potential interest for food companies and policymakers.

## 9. Challenges in the Field of Meat-Alternative Protein Sources

In this field, open challenges may include technological barriers, nutritional and safety issues, sensory acceptability, assessment of real sustainability gain, lack of regulatory framework, and cultural constraints. Whereas technological barriers, nutritional issues, and improvement of sensory traits may crosswise regard all the alternative protein sources treated in this review, lack of regulatory framework and overall acceptability are specific to peculiar sources. For vegetables and mixed vegetable/milk foods, where legal barriers do not exist, the main challenge for researchers is to improve sensory, nutritional, and healthy traits. We presented many cases of traditional fermented vegetable-based foods, which can represent both an inspiration for innovation and an already available tool to modify the diet of most Western consumers. On the opposite side, the ongoing trend for consumers living in developing countries consists of their disaffection with their traditional food and their shift to the Western diet. This could cause a loss of the biodiversity and cultural heritage represented by all those traditional, protein-rich, fermented foods. With this regard, the current challenges are the need to drive fermentation with selected microorganisms and, especially, the usefulness of informing Western consumers about the availability of protein-rich foods that have been for so long in the human diet.

Notwithstanding, several filamentous fungi, and a minor part of (micro)algae and insects, have been part of the diet of some human populations for so long, such food items have to face with cultural constraints in the Western countries. To overcome this challenge, thus offering an additional meat-alternative, it is essential: (i) to increase knowledge about safety, nutrition and, where possible, health-beneficial traits; and (ii) to promote information campaigns that would help to increase consumers’ acceptance [246]. Even before facing consumers’ acceptability, most microbial biomasses and insects must fulfil all the requisites needed to be framed in the regulations on novel foods. Although to a lesser extent, also legumes have to face with acceptability barriers. Indeed, although they often require a lower degree of technological innovations and allow higher sustainability gains, compared to other alternative protein sources, they are considered as “food for the poorest”. This causes a lack of efficient supporting activities exerted by stakeholders’ coalitions. Also in this case, public information campaigns, such as the one promoted by FAO for 2016 as “International Year of the Pulses”, help to fight against this bias [15].

## 10. Future Perspectives

Future actions of research and promotion of meat-alternative protein sources almost directly stem from the challenge analysis shown in the previous paragraph. Here, we would just point out that much work can be implemented on deeper knowledge about protein-rich vegetable resources, which are still under-exploited, but able to adapt to the pedo-climatic scenario resulting from the global warming. To combine flours from different vegetable grains and selected fermenting microorganisms would quickly increase food items’ nutritional value and sensory traits, thus fostering their use as meat alternatives. In addition, we firmly believe that the use of waste and byproducts from agriculture and food processing to cultivate yeasts, filamentous fungi, microalgae, and other microorganisms, as well as to breed insects, deserves further studies, because it allows sustainability gains. Finally, research aiming to correlate consumption of alternative protein sources and benefits to human health would surely increase consumers’ attention towards a more sustainable diet.

## Figures and Tables

**Table 1:** Energy (kcal/100 g) and nutrients content of filamentous fungi-based foods compared to some foods of vegetable and animal origin commonly used as sources of dietary proteins (data adapted from [22,52,58]).

Origin	Food	Energy	Proteins ^a^	Carbohydrates ^a^	Lipids ^a^	Saturated Fatty acid ^a^	Fiber ^a^	Vitamins (μg/100 g)			Mineral (mg/100 g)					
								B6	B9	B12	Ca	P	Fe	Mg	Zn	K
Fungal	Mycoprotein *	85	11	3	2.9	0.7	6	100	114	0.72	48	290	0.4	49	7.6	71
	Shiitake (cooked)	55	1.6	12.3	0.2	0.1	N	N	N	N	3	29	0.4	14	N	12
Vegetable	Tofu, soybean (steamed)	73	8.1	0.7	4.2	N	N	70	15	N	N	95	1.2	23	0.7	63
	Chickpea (re-heated)	129	8.4	18.3	3	0.29	7.1	380	35	N	48	141	1.9	44	1.1	281
Animal	Chicken breast (casseroled)	160	28.4	N ^b^	5.2	29.6	0.9	360	6	N	9	210	0.5	25	1.1	270
	Beef mince (stewed)	209	21.8	N	13.5	47.5	N	170	5	0.8	11	93	0.83	11	2.1	163

* Average values; **^a^** Expressed as g/100 g; ^b^ N = Negligible.

**Table 2:** Substrate of cultivation and protein content (%) of different fungal species (data adapted from [25]).

Species	Substrate	Protein Content
*Aspergillus flavus*	Rice bran	10
*Aspergillus niger*	Apple pomace	17–20
	Banana wastes	18
	Rice bran	11
	Stick water	49
	Potato starch processing waste	38
	Waste liquor	50
*Aspergillus ochraceus*	Rice bran	10
*Aspergillus oryzae*	Rice bran (deoiled)	24
*Neurospora* (*Chrysonilia*) *sitophila*	Lignin	39
*Cladosporium cladosporioides*	Rice bran	10
*Fusarium semitectum*	Rice bran	10
*Monascus ruber*	Rice bran	10
*Penicillium citrinum*	Rice bran	10
*Pleurotus floridanus*	Wheat straw	63
*Trichoderma harzianum*	Cheese whey filtrate	34
*Trichoderma viride*	Citrus pulp	32

**Table 3:** Gross chemical composition (%, w w^−1^) of several species of microalgae.

Species	Proteins	Carbohydrates	Lipids	Ashes	Ref.
*Arthrospira platensis* *	70.9	18.8	9.6	–	[83]
*Spirulina maxima*	80	0.6	7.6	11.6	[88]
*Spirulina* sp. *LEB 18* *	53.6–62.9	5.7–10.2	12–11	10.2–23.7	[83]
*Aphanizomenon flosaquae* *	62	23	3	–	[89]
*Heterochlorella luteoviridis* *	13.8	63.1	9.9	–	[90]
*Chlorella pyrenoidosa* *	31.5	12.9	30.5	–	[91]
*Chlorella vulgaris* *	51.0–58.0	12–17	14–22	–	[89]
*Chlamydomonas reinhardtii* *	48	17	21		[89]
*Odontella aurita* *	25.0	66.1	14.5	–	[92]
*Tetraselmis chuii* *	35–40	30–32	5–8	14–16	[93]
*Tetraselmis CTP4*	40.5–42.7	46.5–41.2	4.9–5.6	7.5–8.2	[94]
*Dunaliella salina*	15.6–23.5	6.0–4.8	60.8–68.3	–	[95]
*Nannochloropisis oculata*	57	8	32	–	[89]
*Tisochrysis lutea*	42.9	8.6	27.9	9.7	[89]
*Haematococcus pluvialis*	48	27	15	–	[89]
*Scenedesmus obliquus*	43.1	16.4	10.7	20	[89]

* Microalgae inserted in the EU Novel Food Catalogue.

**Table 4:** Amino acid profile (g per 100 g of protein) of different microalgae compared to soybean and FAO/WHO values standard for essential amino acids for children aged two-five years.

Species	Ile ^§^	Leu	Val	Lys	Phe	Tyr	Met	Cys	Trp	Thr	Ala	Arg	Asp	Glu	Gly	His	Pro	Ser	Ref.
Arthrospira platensis *	6.7	9.8	7.1	4.8	5.3	5.3	2.5	0.9	0.3	6.2	9.5	7.3	11.8	10.3	5.7	2.2	4.2	5.1	[83]
*Spirulina maxima*	6.0	8.0	6.5	4.6	4.9	3.9	1.4	0.4	1.4	4.6	6.8	6.5	8.6	12.6	4.8	1.8	3.9	4.2	[88]
*Spirulina* sp. LEB 18 *	4.4	8.0	4.6	2.9	5.7	3.2	1.6	0.47	2.5	4.9	6.5	4.9	9.2	10.7	5.2	2.7	4.0	4.3	[83]
*Aphanizomenon* sp	2.9	5.2	3.2	3.5	2.5	-	0.7	0.2	0.7	3.3	4.7	3.8	4.7	7.8	2.9	0.9	2.9	2.9	[89]
Heterochlorella luteoviridis *	1.8	8.1	2.9	8.7	5.4	2.7	1.8	0.4	0.6	5.2	11.1	5.6	0.3	1.3	9.6	1.8	5.5	6.8	[90]
Chlorella pyrenoidosa *	6.2	3.4	5.2	8.1	3.8	1.2	3.3	2.8	n.d.	3.4	5.1	5.9	8.1	7.8	9.8	1.6	n.d.	2.8	[91]
Chlorella vulgaris *	3.8	8.8	5.5	8.4	5.0	3.4	2.2	1.4	2.1	4.8	7.9	6.4	9.0	11.6	5.8	2.0	4.8	4.1	[89]
Chlamydomonas reinhardtii *	1.7	6.9	3.0	8.1	5.0	3.1	2.0	0.3	0.3	4.2	10.5	9.2	0.4	0.7	8.0	2.1	4.6	6.2	[89]
Tetraselmis chuii *	3.5	7.5	4.9	5.7	4.8	3.1	2.5	2.9	2.4	4.1	6.1	9.6	14.4	12.3	6.7	1.6	3.7	4.3	[93]
*Tetraselmis CTP4*	1.1	2.2	1.5	1.7	1.4	0.8	0.6	0.3	0.4	1.3	2.0	1.7	2.9	3.6	1.6	0.1	1.3	1.2	[94]
*Dunaliella salina*	4.0	9.6	7.2	6.0	6.9	4.9	2.8	1.6	0.2	5.2	11.0	8.2	9.6	12.4	8.7	1.7	5.2	4.8	[95]
*Haematococcus pluvialis*	0.5	1.2	0.6	0.7	0.6	0.4	0.1	–	n.d.	0.6	1.3	0.7	1.4	1.9	0.9	–	–	0.9	[89]
*Scenedesmus obliquus*	3.6	7.3	6.0	5.6	4.8	3.2	1.5	0.6	0.3	5.1	9.0	7.1	8.4	10.7	7.1	2.1	3.9	3.8	[89]
Soybean	5.3	7.7	5.3	6.4	5.0	3.7	1.3	1.9	1.4	4.0	5.0	7.4	1.3	19.0	4.5	2.6	5.3	5.8	[96]
FAO/WHO	2.8	6.6	3.5	5.8	–	–	–	–	1.1	3.4	–	–	–	–	–	–	1.9	–	[97]

* Microalgae inserted in the EU Novel Food Catalogue. ^§^ Ile—isoleucine, Leu—leucine, Val—valine, Lys—lysine, Phe—phenylalanine, Tyr—tyrosine, Met—methionine, Cys—cysteine, Trp—tryptophan, Thr—threonine, Ala—alanine, Arg—arginine, Asp—aspartate, Glu—glutamate, Gly—glycine, His—histidine, Pro—proline, Ser—serine.

**Table 5:** Gross chemical composition (%, w w^−1^) of cereals, pseudocereals and legumes.

Species	Proteins	Limiting EAA *	Carbohydrates	Lipids	Fiber	Ashes	Ref.
Barley	9.9–11.60	Met	77.7	1.2–1.9	15.2–15.6	1.6–2.6	[116,125,126]
Rye	8.8–11.4	Cys, Met	60.7	1.7–2.5	12.9–13.2	2.02	[125,126,127]
Triticale	12.3	Met	nr ^¥^	1.74	18.1	2.33	[125]
Spelt	14.6	Lys	53.9	2.4	10.7	nr	[126,128]
Maize	9.4–10.60	Cys	74	4.7	7.3	nr	[116,126,127]
Rice	7.1–15	Trp	80.0	0.7–20	1.3–11	1.35–9.9	[127]
Millet	9.5–11.7	Lys	73	4.2	1.8–8.5	1.17	[125,126]
Sorghum	10.5–12.6	Cys, Met	75	2.2–3.3	6.3–12.1	2.15	[116,125,127]
Oat	8.8–17	Trp, Cys	66.3	4.9–6.9	11.25–11.6	nr	[116,125,127]
Buckwheat	12.5–14.8	Trp	58.9	2.1–3.6	8.3–29.5	2.1	[116,127]
Amaranth	14.5–16.5	Trp	61.4	5.7–10.2	8.8–20.6	2.5	[116,126]
Quinoa	13–14.5	Trp	64.2	5.2–7.2	7.2–14.2	2.9	[116,126]
Pea	15.3–21.9	Trp	52.5	2.34–7.3	10.4–30.7	2.39–3	[119]
Fava bean	21.87–31.2	Met, Cys	nr	2.1–12.45	24.7–31.74	3.13–3.4	[119]
Chickpea	18.5–24.7	Met, Cys	54.0	1.5–6.7	9.88–18.8	3.15–3.7	[116]
Lentil	20.06–25.25	Trp	56.4	2.15–3.27	6.8–33.6	2.0–2.8	[119]
Soybean	34.05–44.53	Met	nr	14.13–22.44	4.2–32.2	3.9–5.05	[116,119]
Lupin	29.5–48.2	Lys, Trp, Met	nr	4.5–10.4	11.6–47.5	3.5–4.9	[116,120]

* Limiting Essential Amino Acid refers to one or more essential amino acids scarcely present in a given cereal/legume. Lys—lysine, Met—methionine, Cys—cysteine, Trp—tryptophan. ^¥^ nr, not reported.

## Data Availability

Not applicable.

## Supplementary Materials

The following supporting information can be downloaded at: https://www.mdpi.com/article/10.3390/foods11142065/s1, Table S1: Gross chemical composition of some mushrooms; Table S2: Composition of fatty acids (% of total fatty acids) of several microalgae.
