# Supplementary material for: Protein Sources Alternative to Meat: State of the Art and Involvement of Fermentation
Source: Foods. 2022 Jul 12;11(14):2065. doi: 10.3390/foods11142065 (PMC9319875; doi:10.3390/foods11142065)
Supplement: Supplementary file 1 [file foods-11-02065-s001.zip › foods-1747941-supplementary.pdf]

Table S1. Comparison of some mushroom compositions (data adapted from [52–55]). * N=Negligible

Origin	Water (%)	Proteins (%)	Lipids (%)	Carbohydrates (%)	Dietary fibre (%)	Minerals (mg/100 g)	Vitamins (mg/100 g)	Energy (kcal in 100 g)
**penny bun**	92–95	4–8	1.6–5.2	1.5–2.5	0.45–1	7–9	25–34	25–35
**champignon**	85–90	2.7–4.8	0.1–0.3	4–6	1–4	590–700	7–8	20–40
**golden chanterelle**	91–95	2.6–3.2	0.7–0.9	3–5	1–2	70–90	5.4–6.2	23–28
***Pleurotus* spp.**	85–95	2.2–2.8	0.2–0.4	4–6	3–6	930–990	33.2–65	28–32
**Caesar's mushroom**	88–93	2–3	0.2–0.4	N *	2	110–120	7–9	11.14
**Truffles**	85	4.5	0.09–2.3	0.17–0.36	8–15	99–115	3.4.2	37–41.1

Table S2. Fatty acids (% of total fatty acids) profile of different microalgae.

Species	C16:0	C18:0	∑ SFA	C16:1	C18:1	∑ MUFA	C18:2 LA	C18:3n-3 ALA	C18:4n-3	C20:5n-3 EPA	C22:6n-3 DHA	∑ PUFA	Ref.
Arthrospira platensis	47.7	12.5	60.1	-	6.3	6.3	10.4	-	-	-	-	10.4	[83]
Spirulina maxima	57.0	2.1	59.1	2.2	6.0	8.2	16.0	1.3				32.7	[88]
Spirulina sp LEB 18	45.4	3.2	48.6	4.1	8.1	12.2	15.5	13.1				28.6	[83]
*Aphanizomenon flosaquae**	36.6	2.7	39.3	11.9	6.7	18.6	7.4	22.3	0.65	0.1		30.4	[89]
*Heterochlorella luteoviridis**	40.9	4.8	45.7	1.7	4.6	6.3	18.5	12.4		2.6	1.0	34.5	[90]
Chlorella pyrenoidosa	22.2	0.9	23.1	0.3	1.2	1.5	13.34	48.2		5.4		66.9	[91]
Chlorella vulgaris	20.2	3.0	23.2	0.7	12.0	12.7	-	18.3	-	0.9	0.5	19.6	[89]
Chlamydomonas mexicana	35.1	1.1	36.2	1.0	6.7	7.7	40.9	14.3	-	-	-	55.2	[89]
Odontella aurita	13.7	2.2	15.9	22.5	10.1	32.6	0.7	0.8	1.3	25.6	3.8	32.2	[92]
*Schizochytrium sp.**	32.2	24.0	56.2	3.8	1.1	4.9				1.3	23.2	24.4	[98]
Tetraselmis chui	0.3	3.1	3.4	3.6	9.8	13.4	4.6	25.2	6.1	8.0			[93]
Tetraselmis sp. CTP4	15.3	0.5	15.8	1.9	11.8	13.7	5.3	18.8	4.9	3.7	-	32.7	[94]
Dunaliella salina	9.8	3.1	12.9	14.7	8.0	22.7	4.9	12.9	-	-	15.4	33.1	[89]
Nannochloropsis oculata	18.4	-	18.4	26.1	3.0	29.1	6.2	-	-	34.0	-	40.2	[89]
Tisochrysis lutea	14.4	0.3	14.7	20.5	15.2	35.7	12.1	-	-	2.8	12.7	27.5	[89]
Haematococcus pluvialis	4.4	2.2	6.6	0.3	16.1	16.4	7.0	21.2		3.5		31.7	[89]
Scenedesmus sp.	20.5	0.8	21.2	19.0	0.9	19.9	10.3	39.3	-	-	-	49.6	[89]

* Microalgae inserted in the EU Novel Food Catalogue.
